# Heterozygous *DHTKD1* Variants in Two European Cohorts of Amyotrophic Lateral Sclerosis Patients

**DOI:** 10.3390/genes13010084

**Published:** 2021-12-29

**Authors:** Alma Osmanovic, Isabel Gogol, Helge Martens, Maylin Widjaja, Kathrin Müller, Olivia Schreiber-Katz, Friedrich Feuerhake, Claus-Dieter Langhans, Gunnar Schmidt, Peter M. Andersen, Albert C. Ludolph, Jochen H. Weishaupt, Frank Brand, Susanne Petri, Ruthild G. Weber

**Affiliations:** 1 Department of Human Genetics, Hannover Medical School, 30625 Hannover, Germany; dr.almaosmanovic@gmail.com (A.O.); isabel.gogol@stud.mh-hannover.de (I.G.); martens.helge@mh-hannover.de (H.M.); maylinwidjaja@web.de (M.W.); schmidt.gunnar@mh-hannover.de (G.S.); brand.frank@mh-hannover.de (F.B.); 2 Department of Neurology, Hannover Medical School, 30625 Hannover, Germany; schreiber-katz.olivia@mh-hannover.de; 3 Essen Center for Rare Diseases (EZSE), University Hospital Essen, 45147 Essen, Germany; 4 Department of Neurology, University of Ulm, 89070 Ulm, Germany; kathrin.mueller@uni-ulm.de (K.M.); albert.ludolph@rku.de (A.C.L.); Jochen.Weishaupt@medma.uni-heidelberg.de (J.H.W.); 5 Department of Neuropathology, Institute of Pathology, Hannover Medical School, 30625 Hannover, Germany; feuerhake.friedrich@mh-hannover.de; 6 GCMS Laboratory, Dietmar Hopp Metabolic Center, University Children’s Hospital, 69120 Heidelberg, Germany; Claus-Dieter.Langhans@med.uni-heidelberg.de; 7 Department of Clinical Sciences, Neurosciences, Umeå University, 90185 Umeå, Sweden; peter.andersen@umu.se; 8 Division for Neurodegenerative Diseases, Department of Neurology, Medical Faculty Mannheim, University of Heidelberg, 68167 Mannheim, Germany

**Keywords:** amyotrophic lateral sclerosis, *DHTKD1*, neurodegeneration, Charcot-Marie-Tooth disease type 2, 2-aminoadipic and 2-oxoadipic aciduria, lower motor neuron, whole-exome sequencing

## Abstract

Amyotrophic lateral sclerosis (ALS) is a fatal neurodegenerative disorder characterized by progressive upper and lower motor neuron (LMN) loss. As ALS and other neurodegenerative diseases share genetic risk factors, we performed whole-exome sequencing in ALS patients focusing our analysis on genes implicated in neurodegeneration. Thus, variants in the *DHTKD1* gene encoding dehydrogenase E1 and transketolase domain containing 1 previously linked to 2-aminoadipic and 2-oxoadipic aciduria, Charcot-Marie-Tooth (CMT) disease type 2, and spinal muscular atrophy (SMA) were identified. In two independent European ALS cohorts (*n* = 643 cases), 10 sporadic cases of 225 (4.4%) predominantly sporadic patients of cohort 1, and 12 familial ALS patients of 418 (2.9%) ALS families of cohort 2 harbored 14 different rare heterozygous *DHTKD1* variants predicted to be deleterious. Four *DHTKD1* variants were previously described pathogenic variants, seven were recurrent, and eight were located in the E1_dh dehydrogenase domain. Nonsense variants located in the E1_dh domain were significantly more prevalent in ALS patients versus controls. The phenotype of ALS patients carrying *DHTKD1* variants partially overlapped with CMT and SMA by presence of sensory impairment and a higher frequency of LMN-predominant cases. Our results argue towards rare heterozygous *DHTKD1* variants as potential contributors to ALS phenotype and, possibly, pathogenesis.

## 1. Introduction

Amyotrophic lateral sclerosis (ALS) is a progressive neurodegenerative disorder characterized by upper and lower motor neuron loss, commonly leading to death due to respiratory paralysis within three to five years after disease onset [1]. ALS is an umbrella term covering a large spectrum of different phenotypes with distinct clinical presentations. Different ALS subtypes can be distinguished depending on the extent of bulbar/spinal upper and lower motor neuron affection with different clinical and prognostic characteristics related to gender and age [2]. A contribution of genetic factors to ALS pathogenesis was suggested by the observation that 5–10% of ALS patients have a familial predisposition, and that relatives of apparently sporadic ALS patients, representing the vast majority of cases, have an increased ALS risk [3]. Three decades of research have implicated at least 25 genes [1] and more than 120 variants in ALS risk (https://alsod.ac.uk/ accessed on 21 October 2021). Additionally, certain genetic aberrations have been reported to modify the ALS phenotype, suggesting that genetic factors also shape the clinical presentation of ALS [4]. Moreover, some of the genes identified in ALS patients are also causative for other neurodegenerative disorders, such as *C9orf72* and *TARDBP* for frontotemporal dementia (FTD) and Parkinson’s disease, and *SPG7* and *SPG11* for hereditary spastic paraplegia, indicating shared pathomechanisms [4,5,6,7]. In fact, it appears as though most, if not all ALS genes may be pleiotropic [8].

Accordingly, in this study, in search of genes implicated in neurodegeneration as potential novel ALS-associated genes, we identified rare heterozygous *DHTKD1* variants in ALS patients. Pathogenic variants in *DHTKD1* encoding dehydrogenase E1 and transketolase domain containing 1 were previously described in patients with autosomal recessive 2-aminoadipic and 2-oxoadipic aciduria (AMOXAD), a rare metabolic disorder also characterized by hypotonia, delayed psychomotor development and seizures [9,10], autosomal dominant Charcot-Marie-Tooth disease type 2 (CMT2) [11], and autosomal recessive infantile-onset spinal muscular atrophy (SMA) with cognitive delay [12]. Here, we aimed at investigating the frequency as well as genotype–phenotype correlations of rare *DHTKD1* variants in two independent ALS cohorts.

## 2. Materials and Methods

### 2.1. Patients

Cohort 1 consisted of 225 unrelated ALS patients of central European ethnicity (133 males, 92 females; 8 with familial ALS, 217 with sporadic ALS) recruited at the motor neuron disease clinic of the Department of Neurology at Hannover Medical School, Hannover, Germany. All patients were examined by a neurologist specialized in motor neuron diseases and subdivided into one of eight ALS subtypes (upper motor neuron (UMN)-dominant ALS, bulbar phenotype, flail arm syndrome, flail leg syndrome, respiratory phenotype, progressive muscular atrophy (PMA), lower motor neuron (LMN)-dominant ALS, and classic (Charcot) ALS) [2]. Extensive clinical workup including magnetic resonance imaging, cerebrospinal fluid analysis, electromyography (EMG), and nerve conduction studies (NCS) was performed at first examination. UMN-dominant ALS was defined by clinically predominant UMN signs at disease onset but development of clinically progressive LMN signs within 2–4 years, thereby distinguishing these cases from primary lateral sclerosis (PLS), according to the recently renewed diagnostic criteria for PLS [13]. While PLS can be considered as part of the ALS spectrum [14], cases of probable or definite PLS [13] were not included into our patient cohort. In cases with absence of UMN signs, the diagnosis of PMA was made after careful exclusion of disease mimics, including search for conduction blocks by extensive NCS and cerebrospinal fluid analysis to rule out immune-mediated neuropathies, genetic testing for deletions or point mutations in the *SMN1* gene or expansion of the CAG repeat in the androgen receptor gene, and/or muscle biopsies [2]. Disease progression was measured using the Revised ALS Functional Rating Scale (ALSFRS-R) [15], the progression rate was calculated as previously described [16]. 

Cohort 2 consisted of 486 familial ALS patients from 418 unrelated families collected in Germany and Sweden [17], thus carrying a comparable genetic background as cohort 1.

### 2.2. Whole-Exome and Targeted DHTKD1 Sequencing 

In cohort 1, extraction of genomic DNA was performed from whole blood using the QIAamp DNA Blood Maxi Kit (Qiagen, Hilden, Germany). Whole-exome sequencing (WES) on leukocyte DNA of 46 ALS patients and 148 control individuals not affected by a neurologic disease was performed using the Agilent SureSelect Human All Exon v4 or v5 + UTR Target Enrichment System (both Agilent Technologies, Santa Clara, CA, USA) or the IDT xGen Exome Research Panel (Integrated DNA Technologies, Coralville, IA, USA) on an Illumina HiSeq 2000 (Illumina, San Diego, CA, USA) or a MGISEQ2000 (MGI Tech, Shenzhen, Guangdong, China) sequencing platform. All samples were sequenced to a mean target coverage of >50×. Sequencing data were aligned to the human reference genome GRCh37/hg19 and analyzed using our in-house workflow and a candidate gene-based strategy with regard to neurodegeneration (Supplementary Table S1) using CLC Genomics Workbench 20 and Clinical Insight Interpret 8.0 (both Qiagen). Targeted sequencing of all coding exons and adjacent splice site regions (±15 base pairs into intron) of the *DHTKD1* gene (NG_033248.1) was done on leukocyte DNA of 179 additional ALS patients by conventional chain termination protocols on a 3130XL Genetic Analyzer (Thermo Fisher Scientific, Waltham, MA, USA) (oligonucleotide sequences are given in Supplementary Table S2). The identified variants were prioritized based on their minor allele frequency (MAF) extracted from the Genome Aggregation Database (gnomAD, v.2.1.1, https://gnomad.broadinstitute.org accessed on 21 October 2021) and in silico prediction of their pathogenicity using MutationTaster (http://www.mutationtaster.org accessed on 21 October 2021), Polyphen-2 (http://genetics.bwh.harvard.edu/pph2/ accessed on 21 October 2021), SIFT and PROVEAN (http://provean.jcvi.org/ accessed on 21 October 2021). *DHTKD1* variants were classified according to the guidelines of the American College of Medical Genetics and Genomics and the Association for Molecular Pathology (ACMG/AMP) [18]. 

In cohort 2, *DHTKD1* variants were extracted from a WES dataset generated as previously described [17], although only *DHTKD1* variants from patients without a pathogenic variant in a known ALS gene were used in this study.

Nucleotide numbering of the identified variants reflects the nucleotide position in the coding sequence of human *DHTKD1* mRNA (NM_018706.6).

### 2.3. Metabolic Analysis of Urine and Plasma of DHTKD1 Variant Carrier VALS164

The 2-aminoadipate and 2-oxoadipate levels in urine and plasma of *DHTKD1* variant carrier VALS164 were determined by the GCMS Laboratory, Heidelberg, Germany. Urine analysis was performed as previously described [19,20] with some modifications. A urine volume equivalent to 1 µmol creatinine was acidified with hydrochloric acid and extracted twice with ethyl acetate. After removal of the solvent, the residue was derivatized with N-methyl-N-trimethylsilylheptafluorobutyramide (Macherey-Nagel, Düren, Germany). The resulting trimethylsilyl derivatives were analyzed using the single quadrupole mass spectrometer DSQ II (Thermo Fisher Scientific) coupled to the gas chromatograph TRACE GC (Thermo Fisher Scientific). The mass spectrometer was run in the full scan mode (m/z 50 to m/z 650) with electron impact ionization. Gas chromatographic separation was achieved on a capillary column (DB-5MS, 30 m × 0.25 mm; film thickness: 0.25 µm; J&W Scientific, Folsom, CA, USA) using helium as a carrier gas. A volume of 1 µL of the derivatized sample was injected in splitless mode. GC temperature parameters were 80 °C for 2 min, ramp 50 °C per minute to 150 °C, ramp 10 °C per minute to 300 °C. Injector temperature was set to 260 °C and interface temperature to 260 °C. The specific mass m/z 484 was used for quantification of 2-oxoadipate.

The 2-aminoadipate levels were determined in urine and plasma by the EZ:faast amino acid GCMS analysis kit (Phenomenex, Torrance, CA, USA) with some modifications. Based on cation-exchange solid-phase extraction, 2-aminoadipate was measured as a chloroformate derivative by gas chromatography mass spectrometry. The system consisted of a gas chromatograph 7820A coupled to the mass spectrometer 5977 Inert MSD (Agilent Technologies). The capillary column used was DB-5MS, 30 m × 0.25 mm; film thickness: 0.25 µm (J&W Scientific). The temperature programming of the gas chromatograph started at an initial temperature of 60 °C and was increased to 290 °C at 7 °C/min. After a hold time of 2 min, the temperature was further increased to 300 °C at 10 °C/min. Helium was used as carrier gas in the constant pressure mode. The temperature of the MSD transfer line was 290 °C. Inlet was operated using the splitless mode with a temperature of 280 °C. Injection volume was 1 µL of the derivatized sample. Quantification ion for 2-aminoadipate was mass m/z 244.

### 2.4. Histological Analysis of a Muscle Biopsy from DHTKD1 Variant Carrier VALS054

A muscle biopsy of patient VALS054 obtained for differential diagnostic purposes during the initial workup was processed according to standard histological techniques. Sections were stained with hematoxylin-eosin, nonspecific esterase, modified Gomori trichrome and combined cytochrome c oxidase and succinate dehydrogenase (COX/SDH).

### 2.5. Statistical Analysis

Statistical analysis was done using MATLAB and Statistics Toolbox Release 2018b (The MathWorks, Natick, MA, USA). Student’s t-test, Fisher’s exact test or Mann–Whitney U test were used, as applicable; a *p*-value < 0.05 was considered statistically significant.

## 3. Results

### 3.1. Genetic Analysis of Two Independent Cohorts of ALS Patients

**Figure 1:**
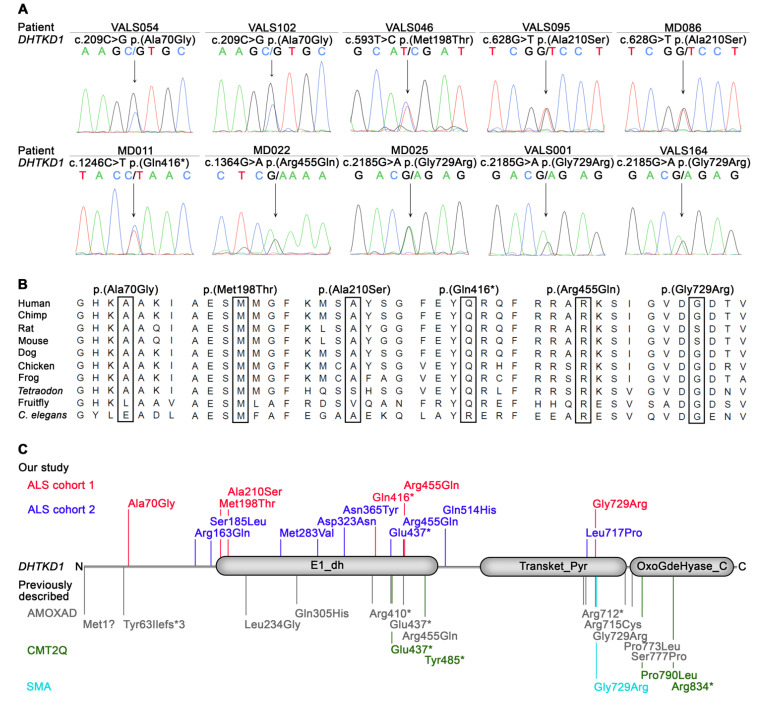
*DHTKD1* variants identified in ALS patients of cohort 1 (**A**,**B**) and cohort 2 in this study, and described in other disorders previously (**C**). (**A**) Electropherograms showing the six different heterozygous rare (MAF ≤ 0.5%) *DHTKD1* variants predicted to be deleterious by at least one in silico prediction tool, i.e., MutationTaster, PolyPhen-2, SIFT, or PROVEAN, detected in leukocyte DNA of 10 sporadic ALS patients of cohort 1 (affected nucleotides are highlighted by an arrow). The c.209C>G p.(Ala70Gly) and c.628G>T p.(Ala210Ser) variants were detected twice, the c.2185G>A p.(Gly729Arg) variant in three ALS patients. (**B**) *DHTKD1* variants identified in ALS patients of cohort 1 affect highly or moderately conserved amino acids, according to Alamut Visual 2.15 (Interactive Biosoftware, Rouen, France). (**C**) Schematic representation of rare *DHTKD1* variants described in ALS patients of this study, i.e., sporadic ALS patients of cohort 1 (red) and familial ALS patients of cohort 2 (blue), and, previously, in the following other diseases: 2-aminoadipic and 2-oxoadipic aciduria (AMOXAD) [9,10,22], Charcot-Marie-Tooth disease type 2Q (CMT2Q) [11,21,23,24], and infantile-onset spinal muscular atrophy (SMA) [12]. The DHTKD1 protein contains three functional domains according to the Pfam database (http://pfam.xfam.org/protein/Q96HY7 accessed on 21 October 2021): (1) dehydrogenase E1 component (E1_dh), (2) transketolase, pyrimidine binding domain (Transket_Pyr), and (3) 2-oxoglutarate dehydrogenase, C-terminal (OxoGdeHyase_C).

To identify novel genes potentially associated with ALS, a pilot WES analysis was performed on 27 genetically unsolved ALS patients of cohort 1. A candidate gene-based strategy was applied prioritizing rare (MAF ≤ 0.5%), non-silent variants not present in in-house controls and predicted to be deleterious in 694 genes associated with neurodegeneration, revealing the *DHTKD1* gene as the only gene harboring such variants in two patients (Supplementary Table S1). In one patient, a heterozygous nonsense variant, *DHTKD1*:c.1246C>T p.(Gln416*), predicted to truncate the encoded protein within the first dehydrogenase domain (E1_dh; Figure 1) was detected. Another patient was found to carry a heterozygous missense variant, *DHTKD1*:c.1364G>A p.(Arg455Gln) (Figure 1, Table 1), previously reported in patients with AMOXAD [10]. As two ALS patients carried rare *DHTKD1* variants in our pilot study and the identified aberrations were loss-of-function and known pathogenic variants, we aimed to determine the frequency of rare *DHTKD1* variants in all 225 patients of cohort 1. Whole-exome and targeted sequencing revealed four additional *DHTKD1* variants, i.e., c.209C>G p.(Ala70Gly), c.593T>C p.(Met198Thr), c.628G>T p.(Ala210Ser), and c.2185G>A p.(Gly729Arg), in eight of 198 further ALS patients (Table 1). Interestingly, the c.2185G>A p.(Gly729Arg) variant, previously described in AMOXAD [9] and SMA [12], was detected in three patients, significantly more frequently than in the gnomAD control data set (3/225 ALS patients of cohort 1 versus 154/59,472 controls; *p* = 0.022, two-sided Fisher’s exact test). In total, six different rare non-silent *DHTKD1* variants predicted to be deleterious were identified in 10 sporadic ALS cases of 225 (4.4%) patients of cohort 1. All were confirmed to be heterozygous by Sanger sequencing (Figure 1A) and affected highly or moderately conserved amino acid residues (Figure 1B). 

Next, we analyzed rare *DHTKD1* variants and their frequency in familial ALS (cohort 2). In cohort 2, nine rare heterozygous *DHTKD1* variants predicted to be deleterious were found in 12 unrelated familial ALS patients of 418 (2.9%) ALS families (Table 2), consisting of eight missense and one nonsense variant. The c.488G>A p.(Arg163Gln), c.847A>G p.(Met283Val) and c.1542G>T p.(Gln514His) variants were detected in two patients each. Two of the variants, i.e., c.1542G>T p.(Gln514His) and c.2150T>C p.(Leu717Pro), were not found in gnomAD database v2.1.1 controls. Five of the variants were located within the first dehydrogenase domain (E1_dh) of the DHTKD1 protein, including the nonsense variant, c.1309G>T p.(Glu437*), predicted to truncate the protein within this domain (Figure 1C). The c.1309G>T p.(Glu437*) variant was previously described in AMOXAD [10] and CMT2Q [21]. The c.1364G>A p.(Arg455Gln) missense variant, previously reported in cases of AMOXAD [10], was identified in cohort 1 and cohort 2 (Figure 1C). 

Of the 14 different *DHTKD1* variants identified in both cohorts, eight were located in the first dehydrogenase domain of the DHTKD1 protein. Notably, loss-of-function variants located in the first dehydrogenase domain of DHTKD1 were significantly more frequent in ALS patients of cohorts 1 and 2 than in gnomAD v2.1.1 controls (2/643 unrelated ALS cases of cohorts 1 and 2 versus 25/51,060 controls; *p* = 0.0442, two-sided Fisher’s exact test).

### 3.2. Clinical Characteristics of Sporadic ALS Patients Carrying DHTKD1 Variants

Sporadic ALS patients with *DHTKD1* variants of cohort 1 (*n* = 10) were available for detailed clinical and electrophysiological phenotyping (Supplementary Table S3). *DHTKD1* variant carriers were almost exclusively from Germany, had a median age of disease onset of 71 years (range: 49–73 years), and the site of onset was mainly spinal (8/10) and less frequently bulbar (2/10). The most frequent symptoms at disease onset were deterioration of fine motor skills or weakness of the hand (6/10) followed by walking difficulties (2/10) or speech difficulties (2/10).

In all sporadic ALS patients carrying *DHTKD1* variants, EMG revealed disseminated signs of acute and chronic denervation. NCS were available in 9/10 *DHTKD1* variant carriers, showing axonal and demyelinating motor neuropathy in 4/9 cases (VALS054, VALS102, VALS095, VALS001), axonal motor neuropathy in 5/9 cases (VALS046, MD011, MD022, VALS164, MD025), and additional sensory neuropathy in 4/9 cases (axonal and demyelinating in VALS054 and VALS095, axonal in MD022 and MD025) with matching clinical signs including pallhypaesthesia, hypaesthesia or reduced sharp/blunt differentiation. Two further *DHTKD1* variant carriers (MD011, VALS164) presented with sensory impairment but normal sensory NCS. No possible underlying causes (e.g., diabetes, alcohol abuse, or chemotherapy) were present in five of the six cases with sensory impairment, whereas patient VALS054 was diagnosed with diabetes mellitus type II (Supplementary Table S3).

To test for signs of AMOXAD in patient VALS164 showing heterozygosity for the c.2185G>A p.(Gly729Arg) variant previously described in AMOXAD patients compound heterozygous for this and another variant [9], the only carrier of an AMOXAD-associated variant from cohort 1 who was still alive, urine and plasma samples of patient VALS164 were subjected to metabolic analysis of 2-aminoadipate and 2-oxoadipate levels. The urine concentration of 2-aminoadipate was close to the upper reference limit, while 2-oxoadipate was hardly detectable in patient VALS164 (Table 3).

**Figure 2:**
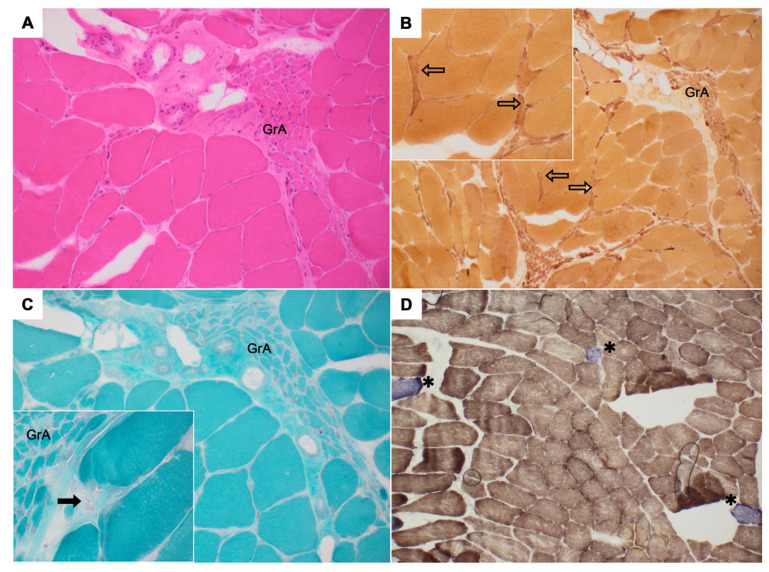
Muscle biopsy of ALS patient VALS054 of cohort 1 diagnosed with the PMA subtype carrying the *DHTKD1*:c.209C>G p.(Ala70Gly) variant. Muscle sections were stained using (**A**) hematoxylin-eosin, (**B**) nonspecific esterase, (**C**) modified Gomori trichrome, and (**D**) combined cytochrome c oxidase and succinate dehydrogenase (COX/SDH). Histopathological analysis revealed a distinctive pattern of neurogenic damage including groups of atrophic muscle fibers (GrA), single atrophic muscle fibers (unfilled arrows), and scattered muscle fiber necrosis (filled arrows). In the COX/SDH stain, up to six fibers per cross section were COX negative (blue fibers denoted by asterisks), suggesting mitochondrial dysfunction.

Patient VALS054 was initially diagnosed with PMA, and a muscle biopsy of the left biceps brachii was obtained for the differential diagnosis of myopathy/myositis. Neuropathological evaluation revealed a distinctive pattern of neurogenic damage including grouped atrophy, single muscle fiber atrophy, and single muscle fiber necrosis upon hematoxylin-eosin, nonspecific esterase, and modified Gomori trichrome staining. In mitochondrial-targeted staining, combined COX/SDH staining displayed reduced or absent COX activity in single muscle fibers (Figure 2).

In direct comparison of *DHTKD1* variant carriers to non-carriers in cohort 1, no significant differences in demographic or individual clinical parameters were identified (Table 4). However, when grouping the ALS subtypes PMA and LMN-dominant ALS together, *DHTKD1* variant carriers more frequently belonged to this grouped subtype with lower motor neuron involvement (2/10, 20% versus 8/215, 3.72% in non-carriers, *p* = 0.066, two-sided Fisher’s exact test), although the difference was not statistically significant. Additionally, there was a trend towards a higher ALSFRS-R progression rate indicating faster disease progression in *DHTKD1* variant carriers compared to non-variant carriers (median ALSFRS-R progression rate/month: 1.17 versus 0.55; *p* = 0.068, Mann–Whitney U test). Furthermore, patients carrying a *DHTKD1* variant more frequently showed severe axonal damage (mean compound motor action potential < 1 mV in median nerve) at initial diagnosis compared to non-variant carriers (3/9, 33.3% versus 14/150, 9.3%; *p* = 0.057, two-sided Fisher’s exact test), although not quite statistically significant.

## 4. Discussion

Since ALS is known for its heterogeneous genetic architecture [26], we aimed at identifying additional genetic factors that may contribute to or modify the phenotype of ALS patients. *DHTKD1* was identified as an interesting candidate gene by WES and a candidate-based approach using a list of genes associated with neurodegeneration (*n* = 694 genes) in a pilot study of ALS patients of cohort 1. Altogether, in cohort 1, 4.4% of cases, i.e., 10 sporadic ALS patients, and in cohort 2, 2.9% of families, i.e., 12 familial ALS patients, were found to carry rare heterozygous *DHTKD1* variants. Collectively, the analysis of both ALS cohorts (*n* = 643 unrelated cases) yielded the identification of 14 different rare *DHTKD1* variants in ALS.

*DHTKD1* is a nuclear gene encoding dehydrogenase E1 and transketolase domain containing 1, a protein involved in the final degradative pathway of L-lysine that is critical for mitochondrial metabolism [9,27]. Accordingly, the diminished *DHTKD1* expression in vitro resulted in mitochondrial dysfunction and increased production of reactive oxygen species [11,25,28]. There is evidence that the alteration of mitochondrial function occurs early and contributes to the pathogenesis of neurodegenerative diseases including ALS and other neuromuscular disorders [29,30]. Consistently, a number of genetic aberrations causing ALS, FTD, and CMT sensorimotor axonal neuropathy were shown to compromise mitochondrial function, e.g., the GGGGCC repeat expansion in *C9orf72* [31], *VCP* (valosin containing protein) mutations [32], *CHCHD10* (coiled-coil-helix-coiled-coil-helix domain containing 10) mutations [33,34], and *DYNC1H1* (dynein cytoplasmic 1 heavy chain 1) mutations [35,36]. Based on these findings, it is conceivable that rare *DHTKD1* variants that affect mitochondrial metabolism may increase ALS risk and be contributors to the ALS phenotype. 

Of the 14 different rare *DHTKD1* variants identified here in 643 unrelated ALS cases, all were predicted to be deleterious according to at least one of four prediction tools, four variants were pathogenic according to the ACMG guidelines [18], eight variants were located in the E1_dh dehydrogenase domain of the DHTKD1 protein, two of which were loss-of-function variants predicted to truncate this important functional domain, and seven variants were detected more than once, providing in silico evidence for the pathogenicity of most of the *DHTKD1* variants described here. Four *DHTKD1* variants detected in our ALS cohorts were previously described in patients with disorders affecting the nervous system, that is autosomal recessive AMOXAD, a metabolic condition characterized by elevated 2-aminoadipate and 2-oxoadipate levels in urine and/or plasma and varying neurological symptoms [9,10], autosomal recessive infantile-onset SMA with cognitive delay [12] and autosomal dominant CMT disease type 2Q (CMT2Q) [21], two neuromuscular disorders, as well as in patients with autosomal dominant eosinophilic esophagitis, sometimes accompanied by muscle weakness [25]. Functional in vitro analyses were previously performed on two of the *DHTKD1* variants identified here and support their pathogenicity. Leandro et al. reported that mutant DHTKD1 harboring the c.1364G>A p.(Arg455Gln) variant was less soluble and enzymatically inactive [37]. Similarly, DHTKD1 containing the c.2185G>A p.(Gly729Arg) variant displayed decreased catalytic efficiency for NADH production when assembled into the 2-oxoadipate dehydrogenase complex (OADHc), of which DHTKD1 is a component [38]. Conversely, recent in vitro data suggest that deficient DHTKD1 activity may be partially compensated by oxoglutarate dehydrogenase (OGDH) [39], arguing against a very severe effect of *DHTKD1* variants. 

In vivo evidence for variant pathogenicity in our study came from a metabolite analysis in ALS patient VALS164 carrying the *DHTKD1*:c.2185G>A p.(Gly729Arg) variant, an AMOXAD disease-causing mutation [9]. Urine levels of 2-aminoadipate, elevated in AMOXAD patients with biallelic *DHTKD1* mutations [9,10,22], were close to the upper reference limit in patient VALS164, although carrying a heterozygous *DHTKD1* variant only. A muscle biopsy performed in ALS patient VALS054 diagnosed with the PMA subtype and carrying the *DHTKD1*:c.209C>G p.(Ala70Gly) variant showed the expected pattern of neurogenic atrophy. Additionally, reduced or missing COX activity was observed in single muscle fibers reflecting heterogeneously distributed mitochondrial dysfunction, potentially related to the ALS phenotype [40,41] and the identified genotype. However, partial COX deficiency may also be associated with other processes, such as ageing. Taken together, epidemiological, in silico, in vitro, and in vivo evidence support a pathogenic effect of 12 of the 14 rare *DHTKD1* variants identified here.

Nonsense variants located in the E1_dh dehydrogenase domain of DHTKD1 were significantly more prevalent in ALS patients of cohorts 1 and 2 of this study compared to controls. Such nonsense variants are predicted to result in a truncated DHTKD1 protein devoid of intact functional domains (Figure 1C). One of these variants, *DHTKD1*:c.1309G>T p.(Glu437*), was previously described in a patient with CMT2, a hereditary motor and sensory axonal neuropathy [21]. A different *DHTKD1* nonsense variant in the same domain, c.1455T>G (p.Tyr485*), was reported in a Chinese pedigree with CMT2 and symmetric muscle wasting, predominant weakness of the distal parts of the lower limbs, decreased or absent deep tendon reflexes, and mild to moderate deep sensory impairment [11]. These data suggest a genetic link between ALS and CMT2, and a phenotypic overlap may be expected in patients harboring variants in the same gene. Consistently, the majority (6/10, 60%) of ALS patients of cohort 1 carrying *DHTKD1* variants suffered from sensory impairment and/or sensorimotor neuropathy, which is normally not a part of the strictly motor neuron phenotype of ALS, whereby an age-dependent effect cannot be excluded in our patients. Moreover, *DHTKD1* variant carriers of ALS cohort 1 were more likely to have predominant LMN involvement than non-variant carriers. Our findings are in line with the fact that a *DHTKD1* variant detected in three ALS patients of cohort 1 was previously described in infantile-onset SMA, another motor neuron disorder characterized by muscular atrophy and weakness due to LMN degeneration, whereby the variant was heterozygous in late-onset ALS and homozygous in infantile-onset SMA [12]. *Dhtkd1*-deficient mice are characterized by progressive weakness and atrophy in the distal limbs with motor and sensory dysfunction aggravated by age, accompanied by decreased nerve conduction velocity [42]. Similarly, nerve conduction studies showed more severe axonal damage in ALS patients with rare *DHTKD1* variants compared to non-variant carriers, although these results only reached borderline significance. Taken together, our data provide evidence that rare *DHTKD1* variants may modify the ALS phenotype and, possibly, contribute to ALS risk. 

In our study, *DHTKD1* variants were identified in both sporadic and familial ALS cases. This is in line with recent literature questioning the utility of distinguishing between familial and sporadic ALS for clinical or even genetic counseling purposes. Pathogenic variants in ALS-related genes have not only been detected in familial but also in apparently sporadic ALS, and, as in familial ALS, the ALS risk is increased in relatives of apparently sporadic ALS patients [3]. Despite the observed similarities of *DHTKD1* variant carriers here, their phenotype does show some heterogeneity. This may be explained by additional genetic and environmental factors in our ALS patients carrying *DHTKD1* variants. Oligogenic inheritance, environmental and lifestyle factors, e.g., smoking and extensive physical exercise, as well as age are discussed to play a role in ALS pathogenesis [26,43,44]. 

## 5. Conclusions

Considering that (i) *DHTKD1* variants and diminished *DHTKD1* expression resulting in reduced enzymatic activity can affect mitochondrial function [9,11,25,28], which is known to be involved in ALS pathogenesis [29,30], (ii) a sensorimotor axonal neuropathy, i.e., CMT2, and a motor neuron disease, i.e., SMA, have been associated with *DHTKD1* variants [11,12,21], (iii) variants in CMT-associated genes have been identified in ALS patients and vice versa in earlier reports, e.g., *CHCHD10* [33,34], *DYNC1H1* [36,45], or *VCP* [46,47], and (iv) this study reports rare heterozygous *DHTKD1* variants in two independent ALS cohorts in 4.4% of cases or 2.9% of families, respectively, with data supporting the pathogenicity of most identified variants, and describes similarities in the ALS phenotype of some *DHTKD1* variant carriers, we propose that *DHTKD1* variants may contribute to and modify the ALS phenotype.

## Figures and Tables

**Table 1:** Rare heterozygous non-silent *DHTKD1* variants predicted to be deleterious identified in sporadic ALS patients of cohort 1 (*n* = 225 ALS patients).

Patient ID	Chromosomal Position ^a^	Exon	Nucleotide Change ^b^	Amino Acid Change ^b^	Reference SNP	MAF (gnomAD Controls ^c^)	Prediction				Previously Reported in	ACMG/AMP Criteria ^d^
							Mutation Taster	PolyPhen-2	SIFT	PROVEAN		
VALS054 VALS102	10:12123525	2	c.209C>G	p.(Ala70Gly)	rs34644609	0.003700	Disease causing	Benign	Tolerated	Neutral	-	Uncertain significance
VALS046	10:12129604	4	c.593T>C	p.(Met198Thr)	-	-	Disease causing	Probably damaging	Damaging	Deleterious	-	Uncertain significance (PM2_moderate; PP3_supporting)
VALS095 MD086	10:12129639	4	c.628G>T	p.(Ala210Ser)	rs146741810	0.002554	Disease causing	Benign	Tolerated	Neutral	-	Uncertain significance (BP4_supporting)
MD011	10:12136158	7	c.1246C>T	p.(Gln416*)	rs200722918	0.00004988	Disease causing	-	-	-	-	Pathogenic (PVS1_very strong; PM1_moderate; PP3_supporting)
MD022	10:12139688	8	c.1364G>A	p.(Arg455Gln)	rs142068634	0.0001394	Disease causing	Probably damaging	Damaging	Deleterious	AMOXAD ^e^ (AR)	Pathogenic (PS1_strong; PS3_strong; PM1_moderate; PP3_supporting)
MD025 VALS001 VALS164	10:12154929	13	c.2185G>A	p.(Gly729Arg)	rs117225135	0.001295	Disease causing	Possibly damaging	Damaging	Deleterious	AMOXAD ^f^ (AR), SMA ^g^ (AR)	Pathogenic (PS1_strong; PS3_strong; PS4_strong; PM1_moderate; PP3_supporting)

Abbreviations: AMOXAD: 2-aminoadipic and 2-oxoadipic aciduria; AR: autosomal recessive; MAF: minor allele frequency; SNP: single nucleotide polymorphism; SMA: spinal muscular atrophy. ^a^ According to GRCh37/hg19. ^b^ According to *DHTKD1* transcript NM_018706.6 and DHTKD1 protein NP_061176.4. ^c^ Genome Aggregation Database v2.1.1 controls. ^d^ [18]. ^e^ [10]. ^f^ [9]. ^g^ [12].

**Table 2:** Rare heterozygous non-silent *DHTKD1* variants predicted to be deleterious identified in familial ALS patients of cohort 2 (*n* = 418 ALS families).

Patient ID	Chromosomal Position ^a^	Exon	Nucleotide Change ^b^	Amino Acid Change ^b^	Reference SNP	MAF (gnomAD Controls ^c^)	Prediction				Previously Reported in	ACMG/AMP Criteria ^d^
							Mutation Taster	PolyPhen-2	SIFT	PROVEAN		
C2-Pt1 C2-Pt2	10:12126716	3	c.488G>A	p.(Arg163Gln)	rs78189904	0.0006568	Disease causing	Benign	Tolerated	Neutral	Eosinophilic esophagitis (AD) ^e^	Uncertain significance (PS1_strong; BP4_supporting)
C2-Pt3	10:12129565	4	c.554C>T	p.(Ser185Leu)	rs149544379	-	Disease causing	Benign	Damaging	Neutral	-	Uncertain significance
C2-Pt4 C2-Pt5	10:12131114	5	c.847A>G	p.(Met283Val)	rs145337285	0.0004113	Disease causing	Benign	Tolerated	Deleterious	-	Uncertain significance (PM1_moderate)
C2–Pt6	10:12131234	5	c.967G>A	p.(Asp323Asn)	rs529235889	0.00001832	Disease causing	Possibly damaging	Damaging	Deleterious	-	Uncertain significance (PM1_moderate; PP3_supporting)
C2-Pt7	10:12133617	6	c.1093A>T	p.(Asn365Tyr)	rs747758630	0.00001828	Disease causing	Probably damaging	Damaging	Deleterious	-	Uncertain significance (PM1_moderate; PP3_supporting)
C2-Pt8	10:12136221	7	c.1309G>T	p.(Glu437*)	rs138884194	0.00004988	Disease causing	-	-	-	CMT2Q ^f^ (AD), AMOXAD ^g^ (AR)	Pathogenic (PVS1_very strong; PS1_strong; PM1_moderate; PP3_supporting)
C2-Pt9	10:12139688	8	c.1364G>A	p.(Arg455Gln)	rs142068634	0.0001394	Disease causing	Probably damaging	Damaging	Deleterious	AMOXAD ^g^ (AR)	Pathogenic (PS1_strong; PS3_strong; PM1_moderate; PP3_supporting)
C2-Pt10 C2-Pt11	10:12139866	8	c.1542G>T	p.(Gln514His)	-	-	Disease causing	Benign	Damaging	Deleterious	-	Uncertain significance (PP3_supporting)
C2-Pt12	10:12150010	12	c.2150T>C	p.(Leu717Pro)	-	-	Disease causing	Probablydamaging	Damaging	Deleterious	-	Uncertain significance (PM1_moderate; PP3_supporting)

Abbreviations: AD: autosomal dominant; AMOXAD: 2-aminoadipic and 2-oxoadipic aciduria; AR: autosomal recessive; CMT2Q: Charcot-Marie-Tooth disease type 2Q; fALS: familial ALS; MAF: minor allele frequency; SNP: single nucleotide polymorphism. ^a^ According to GRCh37/hg19. ^b^ According to *DHTKD1* transcript NM_018706.6 and DHTKD1 protein NP_061176.4. ^c^ Genome Aggregation Database v2.1.1 controls. ^d^ [18]. ^e^ [25]. ^f^ [21]. ^g^ [10].

**Table 3:** Metabolite levels in sporadic ALS patient VALS164 carrying the *DHTKD1*:c.2185G>A p.(Gly729Arg) variant.

Metabolite	Urine (mmol/mol Creatinine)	Plasma (µmol/L)
2-aminoadipate	5.5 (upper limit: 8)	1.9 (upper limit: 6)
2-oxoadipate	0.2 (upper limit: 25)	Not detectable

Metabolite levels and upper limits were determined by the GCMS Laboratory, Heidelberg, Germany.

**Table 4:** Clinical and electrophysiological characteristics of ALS patients from cohort 1 comparing *DHTKD1* variant carriers and non-carriers.

Characteristics	*DHTKD1* Variant Carriers Number (%), Mean ± SD or Median (Range)	*DHTKD1* Non-Variant Carriers Number (%), Mean ± SD or Median (Range)	*p*-Value *	Number of Patients with Available Data (Carriers/ Non-Carriers)
**Sex**				
Male	6 (60)	127 (59.1)	1.0	10/215
Age at onset (years)	71 (49–73)	63 (25–84)	0.272	10/212
Disease duration (years) ^a^	2.50 (1.58–9.08)	2.42 (0.25–19.25)	0.992	10/212
**Site of onset**				
Bulbar	2 (20)	62 (28.8)	0.729	10/215
Spinal	8 (80)	153 (71.2)	0.729	10/215
**ALS subtype**				
Classic (Charcot’s) ALS	6 (60)	143 (66.5)	0.737	10/215
Bulbar	2 (20)	31 (14.4)	0.644	10/215
UMN	0 (0)	3 (1.40)	1.0	10/215
Flail arm	0 (0)	24 (11.16)	0.605	10/215
Flail leg	0 (0)	4 (1.86)	1.0	10/215
Respiratory	0 (0)	2 (0.93)	1.0	10/215
PMA	1 (10)	3 (1.40)	0.167	10/215
LMN	1 (10)	5 (2.33)	0.241	10/215
Grouped PMA and LMN	2 (20)	8 (3.72)	0.066	10/215
**Disease progression**				
Time to wheelchair (years)	1.58 (0.92–8.42)	1.92 (0.25–16.0)	0.911	7/108
Time to NIV (years)	1.04 ± 0.21	2.46 ± 1.45	0.130	2/37
Time to PEG (years)	1.17 (1.17–1.42)	1.83 (0.25–7.58)	0.160	3/51
Weight loss (kg/month)	0.53 (1.67–0)	0.25 (6.33–0)	0.532	10/208
ALSFRS-R progression rate/month ^b^	1.17 (2.17–0.24)	0.55 (7.0–0.04)	0.068	9/188
**Nerve conduction study**				
CMAP in median nerve (mV) ^c^	3.28 ± 2.74	4.44 ± 2.73	0.22	9/150
Severe axonal damage (mean CMAP <1mV in median nerve)	3 (33.3)	14 (9.3)	0.057	9/150
Sensory neuropathy (NCS and/or clinical)	6 (60)	85 (39.5)	0.332	10/215

Abbreviations: ALS: amyotrophic lateral sclerosis; ALSFRS-R: revised amyotrophic lateral sclerosis functional rating scale; CMAP: compound muscle action potential; LMN: lower motor neuron; NCS: nerve conduction study; NIV: non-invasive ventilation; PEG: percutaneous endoscopic gastrostomy; PMA: progressive muscular atrophy; SD: standard deviation; UMN: upper motor neuron. * Significant difference at *p* < 0.05. Comparisons between *DHTKD1* variant carriers and non-variant carriers were made using the two tailed Fisher’s exact test for dichotomous variables, Mann–Whitney U test or Student’s t-test for continuous variables. ^a^ Until last follow-up (VALS054, VALS164), total invasive ventilation (MD086) or death (MD011, MD022, MD025, VALS001, VALS046, VALS095, and VALS102) ^b^ ALSFRS-R progression rate/month: high numbers indicate fast progression, ^c^ CMAP in-house standard for median nerve > 7 mV.

## Supplementary Materials

The following supporting information can be downloaded at: https://www.mdpi.com/article/10.3390/genes13010084/s1, Table S1: Strategy used for the analysis of whole-exome sequencing data of 27 ALS patients of cohort 1, Table S2: PCR primers used for amplification and sequencing of *DHTKD1*, Table S3: Clinical and electrophysiological characteristics of sporadic ALS patients of cohort 1 carrying rare heterozygous *DHTKD1* variants.
