# Supplementary material for: Heterozygous DHTKD1 Variants in Two European Cohorts of Amyotrophic Lateral Sclerosis Patients
Source: Genes (Basel). 2021 Dec 29;13(1):84. doi: 10.3390/genes13010084 (PMC8774751; doi:10.3390/genes13010084)
Supplement: Supplementary file 1 [file genes-13-00084-s001.zip › genes-1511884-supplementary.pdf]

Supplementary Material

Heterozygous *DHTKD1* Variants in two European Cohorts of Amyotrophic Lateral Sclerosis Patients

Alma Osmanovic[1,2,3,†], Isabel Gogol[1,2,†], Helge Martens[1], Maylin Widjaja[1,2], Kathrin Müller[4], Olivia Schreiber-Katz[2], Friedrich Feuerhake[5], Claus-Dieter Langhans[6], Gunnar Schmidt[1], Peter M. Andersen[7], Albert C. Ludolph[4], Jochen H. Weishaupt[4,8], Frank Brand[1], Susanne Petri[2,#,*], Ruthild G. Weber[1,#,*]

[1]Department of Human Genetics, Hannover Medical School, Hannover, 30625, Germany;
[2]Department of Neurology, Hannover Medical School, Hannover, 30625, Germany;
[3]Essen Center for Rare Diseases (EZSE), University Hospital Essen, Essen, 45147, Germany;
[4]Department of Neurology, University of Ulm, Ulm, 89070, Germany;
[5]Department of Neuropathology, Institute of Pathology, Hannover Medical School, Hannover, 30625, Germany;
[6]GCMS Laboratory, Dietmar Hopp Metabolic Center, University Children´s Hospital, Heidelberg, 69120, Germany;
[7]Department of Clinical Sciences, Neurosciences, Umeå University, Umeå, 90185, Sweden;
[8]Division for Neurodegenerative Diseases, Department of Neurology, Medical Faculty Mannheim, University of Heidelberg, Mannheim, 68167, Germany.

[†]These authors contributed equally as first authors to this work.
[#]These authors contributed equally as senior authors to this work.

*Correspondence: Ruthild G. Weber, M.D., Hannover Medical School, Department of Human Genetics, Carl-Neuberg-Str. 1, 30625 Hannover, Germany, Phone: +49 511 532 7751, Fax: +49 511 532 18520, Email: Weber.Ruthild@mh-hannover.de
Susanne Petri, M.D., Hannover Medical School, Department of Neurology, Carl-Neuberg-Str. 1, 30625 Hannover, Germany, Email: Petri.Susanne@mh-hannover.de

Supplementary Table S1. Strategy used for the analysis of whole-exome sequencing data of 27 ALS patients of cohort 1.

Filtering steps	Number of variants
Total variants in exomes (±20 bases into intron) obtained by analyzing leukocyte DNA of 27 ALS patients of cohort 1	89,934
Variants with a read depth of ≥35, a call quality of ≥100, and an allele fraction of ≥45% are retained	66,296
Rare variants (minor allele frequency ≤0.5% in 1000 Genomes Project, gnomAD database, and NHLBI ESP exomes)[a] are retained	7,409
Non-silent variants, i.e. splice site (up to 2 bases into intron), frameshift, stop gained/lost, and missense variants are retained	4,473
Comparison with identically generated exome data of in-house control individuals (n=148), variants not present in controls are retained	1,163
Variants predicted to be pathogenic (by SIFT, PolyPhen-2)[b] in genes affected in at least two of the 27 ALS patients are retained	256
Variants in genes associated with neurodegeneration (n=694)[c] are retained	2 (*DHTKD1:*c.1246C>T p.(Gln416*), *DHTKD1:*c.1364G>A p.(Arg455Gln))

[a]1000 Genomes Project data (https://www.internationalgenome.org/), Genome Aggregation Database (gnomAD; https://gnomad.broadinstitute.org/), NHLBI Exome Sequencing Project (https://evs.gs.washington.edu/EVS/)
[b]SIFT (http://sift.jcvi.org/), PolyPhen-2 (http://genetics.bwh.harvard.edu/pph2/)
[c]According to Clinical Insight Interpret 8.0 (Qiagen, Hilden, Germany)

Supplementary Table S2. PCR primers used for amplification and sequencing of *DHTKD1*.

Exon	Forward primer	Reverse primer
1	5‘-GTTTCGTACTGGGAGCAGGT-3‘	5‘-AGTTACAGAGGATGGACGGG-3‘
2	5‘-GTAAGGTGTCTAGGGCTGTAAGAG-3‘	5‘-GAGAAAACAGGCCTCCTCAGAACT-3‘
3	5‘-CAGGATTATTGAACATGGGGAAGG-3‘<br>5‘-GTTCCAGAATTAACTTGCCAC-3‘	5‘-CTTTATAGGCAGTGAGGCTGACCT-3‘
4	5‘-TCCACTGGAGAAGCTGGC-3‘	5‘-CACCAGAATGAAACCAGCAG-3‘
5	5‘-TTCTTATGCCTCAGCCTCTCTACT-3‘	5‘-AGGCTAAAGCCAGTTGCCTGAA-3‘
6	5‘-GATGCAGTGAGCTGAGATCGTG-3‘	5‘-CTGTGTGCACCAGAGGTTTC-3‘
7	5’-AGCTTTACCGAGGTCACACA-3’<br>5’-TTGATGCAGGAAGTAGGGCT-3’	5’-GAGGATCACTTGAGGCAGG-3’
8	5‘-CTTGACAGTGACTCTCCTTGTCT-3‘<br>5‘-GGCCTCCCAAAATGCTAAGATTG-3‘	5‘-ACAACTGAAAACACCAGACCAACG-3‘<br>5‘-CAGTTTCTAGAACCTAACGACACACA-3‘
9	5‘-GCCTGGCCTGCATTAAATTTTTTG-3‘	5‘-GCTTTAGAACCTCTCTGGTCAATG-3‘
10	5‘-CCTCTCCACTCTTATGCAATCTCA-3‘	5‘-CTCTAGGGGTTACTTGCATTCCAA-3‘
11	5‘-ATGAACGAGAGCAAGGCTCC-3‘	5‘-GCCACACTCCCGTCAATAACA-3‘
12	5‘-AGCATAACAGTTCTAGAAGGTGCA-3‘	5‘-CCTGAGGCATTACGTTTCCAAAGA-3‘
13	5‘-CGCACCGTTCATAAATTCACCT-3‘	5‘-TCGCTTAAACCCAGGAGTCA-3‘
14	5‘-CAAGGCATTGCGTCTATTAGGAC-3‘	5‘-GAGGCGGAGGTTGCATAGAG-3‘
15	5‘-CTGAAGAAGGGTCCCCAGGAATAG-3‘<br>5‘-GGTCTTGATTTCCTGACCTCGTGA-3‘	5‘-CCCGGCCCAAGATTCAATTTTCTA-3‘
16	5‘-GTGAATGTGAATCCCTTTCACACC-3‘	5‘-GAAGAGATTAGAGGGACAGGGCTC-3‘
17	5‘-GGAGCCCTGTCCCTCTAATC-3‘	5‘-GAGGAAGGAGGCCTCTTTTAATGG-3‘

Supplementary Table S3. Clinical and electrophysiological characteristics of sporadic ALS patients of cohort 1 carrying rare heterozygous *DHTKD1* variants.

**Patient**	**Amino acid change**	**Gen-der**	**Country of origin**	**Age of onset** (years)	**Site of onset**	**ALS subtype**[a]	**Disease duration** (years)[b]	**ALSFRS-R progression rate**	**Sensory impairment**	**EMG**	**NCS**	**CMAP in median nerve** (mV)[c]
VALS054	p.(Ala70Gly)	M	Germany	72	S	PMA	>9.08	0.24	Pallhypaesthesia, hypaesthesia	Acute/chronic denervation in UL and LL	Axonal and demyelinating sensorimotor neuropathy	0.98
VALS102	p.(Ala70Gly)	F	Germany	57	S	Classic	1.67	1.63	None	Acute/chronic denervation in UL and LL	Axonal and demyelinating motor neuropathy	1.27
VALS046	p.(Met198Thr)	M	Poland	53	S	Classic	3.25	0.40	None	Acute/chronic denervation in UL and LL	Axonal motor neuropathy	5.15
MD086	p.(Ala210Ser)	M	Germany	49	S	Classic	>1.58	1.17	None	Acute/chronic denervation in UL; acute denervation in LL	-	-
VALS095	p.(Ala210Ser)	M	Germany	73	S	LMN	2.83	-	Pallhypaesthesia	Acute/chronic denervation in UL and LL	Axonal and demyelinating sensorimotor neuropathy	1.30
MD011	p.(Gln416*)	F	Germany	69	B	Bulbar	1.83	0.57	Paresthesia	Acute/chronic denervation in LL; acute denervation in UL	Axonal motor neuropathy	4.92
MD022	p.(Arg455Gln)	M	Germany	72	S	Classic	3.33	1.71	Pallhypaesthesia, reduced sharp/blunt differentiation	Acute/chronic denervation in UL and LL	Axonal sensorimotor neuropathy	0.93
VALS001	p.(Gly729Arg)	F	Germany	70	S	Classic	2.17	1.26	None	Acute/chronic denervation in UL and LL	Axonal and demyelinating motor neuropathy	0.33
VALS164	p.(Gly729Arg)	F	Germany	71	B	Bulbar	>3.08	2.17	Pallhypaesthesia, hypaesthesia, and reduced sensation of cold in the feet	Chronic denervation in UL and LL; acute denervation in thorax and tongue	Axonal motor neuropathy	8.08
MD025	p.(Gly729Arg)	M	Germany	73	S	Classic	2.17	0.77	Pallhypaesthesia	Acute/chronic denervation in UL and LL	Axonal sensorimotor neuropathy	6.55

Abbreviations: ALS: amyotrophic lateral sclerosis; ALSFRS-R: revised ALS functional rating scale; B: bulbar; CMAP: compound muscle action potential; EMG: electromyography; F, female; LL: lower limb; LMN: lower motor neuron subtype; M: male; NCS: nerve conduction study; PMA: progressive muscular atrophy; S: spinal; UL: upper limb; -, not available.

[a]Chiò et al. 2011

[b]Until last follow-up (VALS054, VALS164), total invasive ventilation (MD086), or death (MD011, MD022, MD025, VALS001, VALS046, VALS095, and VALS102)

[c]Mean CMAP in median nerve (right and left) at time of initial diagnosis, CMAP in-house standard for median nerve >7mV

References

Chiò, A.; Calvo, A.; Moglia, C.; Mazzini, L.; Mora, G. Phenotypic heterogeneity of amyotrophic lateral sclerosis: a population based study. *J. Neurol. Neurosurg. Psychiatry* **2011**, *82*, 740-746.
